# *De novo* CD5-positive diffuse large B-cell lymphomas show high specificity for cyclin D2 expression

**DOI:** 10.1186/1746-1596-8-81

**Published:** 2013-05-15

**Authors:** Takuro Igawa, Yasuharu Sato, Katsuyoshi Takata, Noriko Iwaki, Takehiro Tanaka, Naoko Asano, Yoshinobu Maeda, Yorihisa Orita, Naoya Nakamura, Shigeo Nakamura, Tadashi Yoshino

**Affiliations:** 1 Department of Pathology, Okayama University Graduate School of Medicine, Dentistry and Pharmaceutical Sciences, 2-5-1 Shikata-cho, Okayama 700-8558, Japan; 2 Department of Pathology, Okayama University Hospital, Okayama, Japan; 3 Department of Clinical Laboratory, Nagoya University Hospital, Nagoya, Japan; 4 Department of Hematology and Oncology, Okayama University Graduate School of Medicine, Dentistry and Pharmaceutical Sciences, Okayama, Japan; 5 Department of Otolaryngology, Head and Neck Surgery, Okayama University Graduate School of Medicine, Dentistry and Pharmaceutical Science, Okayama, Japan; 6 Department of Pathology, Tokai University School of Medicine, Isehara, Japan; 7 Department of Pathology, Nagoya University Hospital, Nagoya, Japan

**Keywords:** Cyclin D2, CD5, Diffuse large B-cell lymphoma

## Abstract

D cyclins positively regulate the cell cycle and mediate the pathogenesis of some lymphomas. Cyclin D1 overexpression is the hallmark of mantle cell lymphoma, whereas cyclins D2 and D3 are reportedly not as specific to certain lymphomas as cyclin D1. In this study, cyclin D2 was found to be overexpressed in 98% of *de novo* CD5-positive diffuse large B-cell lymphomas (DLBCLs) (50/51) and in 28% of CD5-negative DLBCLs (14/51). A statistically significant difference was observed between these two groups (p<0.0001). In contrast, no statistical difference was found in the cyclin D3 expression between CD5-positive (18/51) and CD5-negative (24/51) DLBCLs (p=0.23). Based on these findings, cyclin D2 is therefore considered to be closely associated with *de novo* CD5-positive DLBCLs. This insight may be useful for overcoming the inferior survival of this aggressive lymphoma.

**Virtual slides:**

The virtual slide(s) for this article can be found here: http://www.diagnosticpathology.diagnomx.eu/vs/1382856320966453

## Background

D-type cyclins (D1, D2, and D3) positively regulate the cell cycle and mediate the pathogenesis of some lymphomas [1]. The overexpresison of cyclin D1 due to translocation t (11;14)(q13;q32) serves as a hallmark for the diagnosis of mantle cell lymphoma [1]. Recently, a rare type of cyclin D1-negative mantle cell lymphoma, which overexpresses cyclins D2 or D3, was identified [2]. Cyclins D2 and D3 are also detected in various lymphomas, but they have not been shown to be closely associated with any particular subtype of lymphoma [3].

We recently demonstrated cyclin D2 to be overexpressed in the proliferation centers of chronic lymphocytic leukemia/small lymphocytic lymphoma (CLL/SLL) [4]. CLL/SLL is considered to be a disease caused by the accumulation of CD5-positive B lymphocytes, and proliferating tumor cells are located in the proliferation centers of lymph nodes [5]. The proliferating cells are composed of paraimmunoblastic cells and most of them are in the cell cycle, expressing Ki-67 antigen [5]. These findings suggest that *de novo* CD5-positive diffuse large B-cell lymphomas (CD5^+^ DLBCLs) share the characteristics of actively proliferating cells in CLL/SLL.

*De novo* CD5^+^ DLBCLs account for approximately 10% of all DLBCL cases, and they are characterized by a female predominance, a higher age at diagnosis, and a high degree of central nervous system relapse [6]. *De novo* CD5^+^ DLBCLs are also known to show a significantly poorer survival outcome than CD5-negative DLBCLs (CD5^−^ DLBCLs) under both cyclophosphamide, doxorubicin, vincristine, and prednisone (CHOP) and rituximab-CHOP (R-CHOP) therapy [6,7].

According to these previous data, we sought to clarify the expression patterns of cyclins D2 and D3 in *de novo* CD5^+^ DLBCLs.

## Methods

### Case selection

We studied 51 Japanese patients with *de novo* CD5^+^ DLBCLs diagnosed between 1998 and 2011 at Okayama University, Tokai University, and Nagoya University. The patients included 26 males and 25 females between 32 and 90 years of age (median age 68 years). The examined tissue specimens were obtained from 32 lymph nodes and 19 extranodal sites. The CD5 antigen expression was examined by means of immunohistochemistry of paraffin sections and/or flow cytometry. All samples were immunohistochemically confirmed to be cyclin D1 and sox11 negative [2]. Any samples with a history of other lymphoproliferative disorders were excluded from the study.

As a control group, samples taken from 51 patients with CD5^−^ DLBCLs diagnosed between 1997 and 2011 at Okayama University were also examined. The patients included 27 males and 24 females between 23 and 89 years of age (median age 68 years). The examined tissue specimens were obtained from 42 lymph nodes and 9 extranodal sites. In all cases, the CD5 antigen expression was examined by both immunohistochemistry and flow cytometry.

### Histological examination and immunohistochemistry

The tissue samples were fixed in 10% formalin and embedded in paraffin. The sections (4-μm thick) were stained with H&E. Immunohistochemistry was performed on the paraffin-fixed sections using an automated Bond-max stainer (Leica Biosystems, Melbourne, Australia) and anti-cyclin D2 (polyclonal; 1:150; Proteintech Group Inc., Chicago, IL, USA) and anti-cyclin D3 (DCS-22; 1:10; Progen Biotechnik GmbH, Heidelberg, Germany) antibodies. Based on previous studies, a sample was considered to be positive if ≥20% of the tumor cells were stained [3]. Faint cytoplasmic staining for cyclin D2 without corresponding nuclear staining was not considered positive.

### Statistical analysis

The correlations between the 2 groups were examined by a chi-square analysis. All statistical analyses were carried out with the SPSS software program (version 14.0, SPSS Inc., Chicago, USA).

## Results and discussion

**Figure 1:**
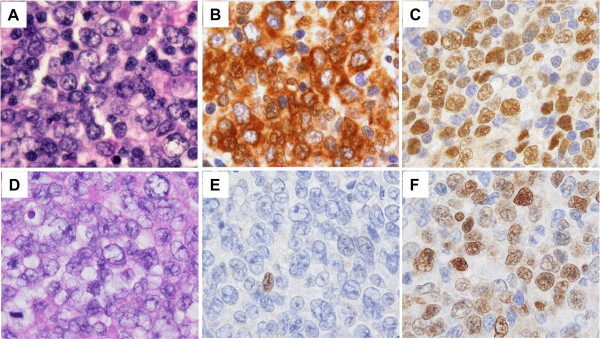
**Cyclins D2 and D3 expression in *de novo *CD5-positive and CD5-negative DLBCLs.** Cyclin D2 expression in *de novo* CD5-positive (**A**, **B**, and **C**) and CD5-negative (**D**, **E**, and **F**) DLBCLs, H&E staining (**A** &**D**), cyclin D2 staining (**B** &**E**), and cyclin D3 staining (**C** &**F**) (×400). Cyclin D2 staining was frequently localized to both the nucleus and the cytoplasm in the *de novo* CD5-positive DLBCLs (**B**). Cyclin D3 showed a crisp nuclear staining pattern in both CD5-positive and negative DLBCLs (**C** &**F**).

In this study, our data showed that cyclin D2 was overexpressed in 98% of *de novo* CD5^+^ DLBCLs (50/51) and in 28% of CD5^−^ DLBCLs (14/51) (Table 1, Figure 1). A statistically significant difference was observed between these two groups (p<0.0001). In contrast, no statistical difference was found in the cyclin D3 expression between CD5-positive (18/51) and CD5-negative (24/51) DLBCLs (p=0.23) (Table 1, Figure 1). Since *de novo* CD5^+^ DLBCLs are typically immunohistochemically negative for cyclin D1 [6], these findings therefore indicate that cyclin D2 is closely associated with *de novo* CD5^+^ DLBCLs.

**Table 1:** **Cyclins D2 and D3 expression in *de novo *CD5**^**+ **^**and CD5**^**− **^**DLBCLs**

	***De novo *CD5**^**+**^**DLBCLs**	**CD5**^**−**^**DLBCLs**	**P**
Cyclin D2	50/51 (98%)	14/51 (28%)	P < 0.0001
Cyclin D3	18/51 (35%)	24/51 (47%)	P = 0.23

Previous studies have examined the cyclins D2 and D3 expression in DLBCLs by immunohistochemistry (Table 2) [3,8-10]. Although the rates of cyclins D2 and D3 positive cases were highly variable in these studies, our findings suggest that cyclin D2-positive DLBCLs comprise the majority of *de novo* CD5^+^ DLBCLs.

**Table 2:** Previous reports on cyclins D2 and D3 expression by immunohistochemistry in DLBCLs

**Reference (reference no.)**		**No. of patients**	**Cut-off line (%)**	**Positive cases (%)**	
				**Cyclin D2**	**Cyclin D3**
Hans CP, et al., 2004 [8]		152	30	13	ND
Hans CP, et al., 2005 [9]		200	30	19	62
Amen F, et al., 2007 [10]		80	10	25	ND
Metcalf RA, et al., 2010 [3]		194	20	49	20
Present study CD5^+^ DLBCLs		51	20	98	35
CD5^−^ DLBCLs		51	20	28	47

ND, not done.

Several studies have shown cyclin D2 to be an independent indicator of an inferior survival in DLBCLs [8-10]. In addition, Lossos et al. identified *CCND2* to be the best predictor of an inferior survival in DLBCLs among the 36 genes associated with their prognosis based on quantitative RT-PCR [11]. These data suggest that cyclin D2 may be associated with the inferior survival observed in *de novo* CD5^+^ DLBCLs. The current gold standard therapy has evolved to include rituximab, and the outcome of DLBCLs has been reported to have dramatically improved [12]. However, both *CCND2* and CD5 have been demonstrated to still be a good indicator of the inferior survival in DLBCLs, even in the era of R-CHOP [7,12]. This fact also suggests an association between cyclin D2 and the poor survival in *de novo* CD5^+^ DLBCLs. Similar to *de novo* CD5^+^ DLBCLs, cyclin D2-positive CD5^−^ DLBCLs may play a role in the negative prognostic impact of cyclin D2 in DLBCLs. In the current study, however, whether or not cyclin D2-positive CD5^−^DLBCLs show a poor survival was not examined. As a result, the prognostic impact of this population therefore still needs to be investigated.

## Conclusions

Cyclin D2 is closely associated with *de novo* CD5^+^ DLBCLs. This insight might be useful for making treatment strategies and improving the survival of this aggressive lymphoma, since cyclin D2 expression is controlled by multiple signaling pathways, such as the NFkB-related pathways [13].

## Consent

Informed consent was obtained from the patient for publication of this report and any accompanying images.

## Abbreviations

DLBCL: Diffuse large B-cell lymphoma; CLL/SLL: Chronic lymphocytic leukemia/small lymphocytic lymphoma; CHOP: Cyclophosphamide, doxorubicin, vincristine, and prednisone; R-CHOP: Rituximab with cyclophosphamide, doxorubicin, vincristine, and prednisone; RT-PCR: Reverse transcription polymerase chain reaction; NFkB: Nuclear factor kappa beta.

## Competing interests

The authors declare that they have no competing interest.

## Authors’ contributions

Conceived and designed the experiments: YS, TI. Performed the experiments: TI, YS. Analyzed the data: YS, TI, TY, KT, NI, TT, YM, NA, YO. Contributed reagents/materials/analysis tools: NN, SN, YM. Wrote the paper: TI, YS, TY. All authors read and approved the final manuscript.
